# Associations Among Different Domains of Quality Among US Liver Transplant Programs

**DOI:** 10.1001/jamanetworkopen.2021.18502

**Published:** 2021-08-09

**Authors:** Craig S. Brown, Seth A. Waits, Michael J. Englesbe, Christopher J. Sonnenday, Kyle H. Sheetz

**Affiliations:** 1 Department of Surgery, University of Michigan, Ann Arbor

## Abstract

**Importance:**

US liver transplant programs have traditionally been evaluated on 1-year patient and graft survival. However, there is concern that a narrow focus on recipient outcomes may not incentivize programs to improve in other ways that would benefit patients with end-stage liver disease.

**Objective:**

To determine the correlation among different potential domains of quality for adult liver transplant programs.

**Design, Setting, and Participants:**

This retrospective cohort study was conducted from 2014 to 2019 among adult liver transplant programs included in the United Network for Organ Sharing and Scientific Registry of Transplant Recipients program-specific reports. Liver transplant programs in the United States completing at least 10 liver transplants per year were included. Data were analyzed from March 2 to August 13, 2020.

**Main Outcomes and Measures:**

The potential domains of quality examined included recipient outcomes (1-year graft and patient survival), aggressiveness (ie, marginal graft use, defined as the rate of use of donors with body mass index [calculated as weight in kilograms divided by height in meters squared] greater than 40, age older than 65 years, or deceased by cardiac death), and waiting list management (ie, waiting list mortality). The correlation among measures, aggregated at the center level, was evaluated using linear regression to control for mean Model for End Stage Liver Disease–Sodium score at organ allocation. The extent to which programs were able to achieve high quality across multiple domains was also evaluated.

**Results:**

Among 114 transplant programs that performed a total of 44 554 transplants, the mean (SD) 1-year graft and patient survival was 90.3% (3.0%) with a total range of 75.9% to 96.6%. The mean (SD) waiting list mortality rate was 16.7 (6.1) deaths per 100 person-years, with a total range of 6.3 to 53.0 deaths per 100 person years. The mean (SD) marginal graft use rate was 15.8 (8.8) donors per 100 transplants, with a total range of 0 to 49.3 donors. There was no correlation between 1-year graft and patient survival and waiting list mortality (β = −0.053; *P* = .19) or marginal graft use (β = −0.007; *P* = .84) after correcting for mean allocation Model for End Stage Liver Disease–Sodium scores. There were 2 transplant programs (1.8%) that performed in the top quartile on all 3 measures, while 4 transplant programs (3.6%) performed in the bottom quartile on all 3 measures.

**Conclusions and Relevance:**

This cohort study found that among US liver transplant programs, there were no correlations among 1-year recipient outcomes, measures of program aggressiveness, or waiting list management. These findings suggest that a program’s performance in one domain may be independent and unrelated to its performance on others and that the understanding of factors contributing to these domains is incomplete.

## Key Points

**Question:**

Are there any correlations among different potential domains of quality among adult liver transplant programs?

**Findings:**

In this cohort study of 114 transplant programs that performed a total of 44 554 transplants, there was no correlation among measures reflecting recipient outcomes (1-year graft and patient survival), program aggressiveness (marginal graft use rate), or waiting list management (waiting list mortality rate). Furthermore, only 2 adult liver transplant programs were found to be in the best performing quartile for all 3 domains concurrently.

**Meaning:**

These findings suggest that institutional-, regional-, or policy-level interventions to improve transplant program performance in one domain of quality may not be associated with deleterious outcomes in improvement in other potentially related domains.

## Teaser

This cohort study examines correlations among different potential domains of quality for US adult liver transplant programs.

## Introduction

Since 1991 the United Network for Organ Sharing (UNOS) and the Scientific Registry of Transplant Recipients (SRTR) have been required to publish regular reports on program-specific survival rates for all solid organ transplants performed in the United States.^1^ While this has increased the regulatory pressure on transplant programs, it has also been associated with improvements in outcomes. For example, since the start of public reporting, 1-year graft and patient survival rates for liver transplant programs improved from approximately 82% in 1992 to 92% in 2020.^2^

Despite improvements in outcomes, there are growing concerns over the singular focus on program-specific recipient survival rates. First, 1-year graft and patient survival rates do not vary much, with 95% of programs rating within 7% of the mean in 2020, a statistic that has not changed substantially over the last 30 years.^2^ This may make it difficult to identify the best and worst performing programs and limit how much can be learned from each for the purpose of quality improvement. Second, the narrow focus on recipient outcomes may detract from opportunities to improve in other domains of quality. For example, there may be value in programs’ use of marginal organs, termed *aggressiveness*, to maximize the number of patients receiving transplants. There may also be value in optimizing outcomes for patients on the waiting list, which reflects how the broader population of patients with liver disease are treated.^3,4^ However, changes in practice to improve quality in one domain may adversely impact performance in the others. At present, little is known about the correlation between potential domains of quality among liver transplant programs.

To address this, we examined the correlations among 1-year graft and patient survival and 2 proxy measures, waiting list management and program aggressiveness, after deceased-donor liver transplantation using aggregated data from UNOS and SRTR. We hypothesized that aggressiveness will be inversely correlated with waiting list mortality and that aggressiveness will be inversely correlated with recipient outcomes, specifically 1-year graft and patient survival. In this study, we evaluated whether performance within each domain was individually associated with performance in the other 2 domains and sought to determine what proportion of US transplant programs excelled in 1, 2, or all 3 domains.

## Methods

### Data Source and Study Population

This cohort study was deemed exempt from approval and informed consent by the institutional review board at the University of Michigan, Ann Arbor, owing to the use of retrospective deidentified data. This study followed the Strengthening the Reporting of Observational Studies in Epidemiology (STROBE) reporting guideline for cohort studies.

From UNOS, we requested and received program-identified data, from January 2014 to December 2019, regarding the proportion and total count of donors with the following characteristics: deceased by cardiac death, age older than 65 years, and body mass index (BMI; calculated as weight in kilograms divided by height in meters squared) greater than 40. These data were merged with SRTR National Center-Level Summary data regarding 1-year graft and patient survival for deceased donor liver transplants, mean Model for End Stage Liver Disease–Sodium (MELD-Na) allocation scores, and program-level aggregated data regarding waiting list mortality extracted from program-specific reports, which are also publicly available on the SRTR website.^2^

All 146 liver transplant programs in the US with data available were included, except for those that met the following exclusion criteria: fewer than 10 liver transplants per year (19 programs) or pediatric-only programs (13 programs). This resulted in a final cohort of 114 liver transplant programs with data available for analysis.

### Outcomes

We measured recipient outcomes using 1-year patient and graft survival. We generated proxy measures for waiting list management quality and program aggressiveness. For waiting list management, we used program-specific 1-year waiting list mortality rates, which are available in the program-specific reports. For program aggressiveness, we calculated the rate of marginal graft use per 100 transplant episodes, which included the use of any graft from a donor of age older than 65 years, with BMI greater than 40, or who was deceased by cardiac death. While no universal definition of aggressive graft use exists, a variety of measures, including age, Centers for Disease Control and Prevention (CDC) classification of increased infectious risk, elevated liver function test results, donation after cardiac death, and elevated BMI. The criteria chosen for this analysis were chosen a priori, because they represent the most common categories of marginal grafts that have been published, with the exception of CDC increased infectious risk classification, which has not been associated with worse outcomes and which recent data suggest is not associated with differential organ use rates.^3,5^ After the creation of these proxy measures of quality, our primary outcomes of interest were the correlation coefficients among these 3 domains of quality. An additional analysis was undertaken to assess the role that donor service area (DSA) competition had in associations among domains of quality. We split the included transplant centers into low competition (contained within a DSA with ≤3 included transplant centers) and high competition (contained within a DSA with >3 included transplant centers) and repeated our analysis assessing the associations with performance across the 3 domains of quality.

### Statistical Analysis

Data were extracted and analyzed between March 2 and August 13, 2020. The purpose of this analysis was to evaluate the association between measures of transplant program quality. The 3 domains included recipient outcomes using mean 1-year graft and patient survival, waiting list management using mean unadjusted waiting list mortality rates, and program aggressiveness using proportion of donors meeting the criteria for marginal graft. Since our data sources report these outcomes yearly, along with the number of patients both on each program’s waiting list and who received transplants during the study period, these data were aggregated over the study period to allow for the calculation of program-specific mean 1-year graft and patient survival, allocation MELD-Na, and waiting list mortality rates for the entire study period.

We report descriptive statistics and completed univariable testing with the Kruskal-Wallis test for nonparametric variables and the analysis of variance test for parametric variables. We assessed correlations between the combinations of each of these 3 domains using linear regression models. All models included mean allocation MELD-Na for the transplant center over the study period as a covariate. STATA statistical software, version 16.1/MP, (StataCorp) was used to perform all statistical analyses. We used a 2-sided approach at the *P* = .05 significance level for all hypothesis testing.

## Results

### Program Characteristics and Quality Measure Variation

A total of 114 transplant programs were included, completing 44 554 liver transplants over the study period. The median (interquartile range [IQR]) annual deceased-donor liver transplant volume was 69 (38-117) donors and the mean (SD) allocation MELD-Na was 29.1 (3.0). Among all programs, mean (SD) 1-year graft and patient survival was 90.3% (3.0%) with a total range of 75.9% to 96.6%. The mean (SD) waiting list mortality rate was 16.7 (6.1) deaths per 100 person-years, with a total range of 6.3 to 53.0 deaths per 100 person years. The mean (SD) marginal graft use rate was 15.8 (8.8) donors per 100 transplants, with a total range of 0 to 49.3 donors. A summary of demographic differences among programs stratified by performance in the domain of recipient outcomes is reported in Table 1.

**Table 1.** Program Characteristics

Characteristic	1-y graft and patient survival quartile			*P* value^**a**^
	Bottom (n = 29)	Middle 2 (n = 57)	Top (n = 28)	
Annual transplant volume, median (IQR)^b^	45 (23-96)	92 (43-121)	54 (34-85)	.049
Region, No. (%)				
1	1 (3.4)	4 (7.0)	1 (3.6)	.52
2	7 (24.1)	5 (8.8)	3 (10.7)	
3	5 (17.2)	8 (14.0)	3 (10.7)	
4	3 (10.3)	7 (12.3)	3 (10.7)	
5	4 (13.8)	5 (8.8)	6 (21.4)	
6	0	2 (3.5)	3 (10.7)	
7	2 (6.8)	8 (14.0)	0	
8	1 (3.4)	4 (7.0)	4 (14.3)	
9	2 (6.8)	4 (7.0)	1 (3.6)	
10	2 (6.8)	5 (8.8)	1 (3.6)	
11	2 (6.8)	5 (8.8)	3 (10.7)	
Allocation MELD-Na, mean (SD)	29.5 (3.4)	28.9 (2.6)	29.1 (3.2)	.62

Abbreviations: IQR, interquartile range; MELD-Na, Model for End Stage Liver Disease–Sodium.

^a^ *P* value generated with the Kruskal-Wallis test for nonparametric variables and the analysis of variance test for parametric variables.

^b^ Calculated as the program-level median annual volume of deceased-donor liver transplants during the study period.

### Correlations Among Each of the Domains of Quality

A summary of differences in the outcomes assessed among programs in each category of quality, stratified by performance, is reported in Table 2. There were no statistically significant correlations among any of the potential domains of quality across quartiles of performance.

**Table 2.** Outcomes by Measure

Outcome	Quartile, Mean (SD)			*P* value^a^
	Bottom	Middle 2	Top	
**1-y graft survival**				
Programs, No.	29	57	28	
Marginal graft use rate^b^	13.8 (8.5)	17.0 (8.0)	15.4 (10.5)	.22
Waiting list mortality rate^c^	18.9 (8.7)	15.8 (4.6)	16.2 (5.2)	.08
1-y graft and patient survival rate, %	86.6 (2.5)	90.5 (1.0)	93.8 (1.1)	<.001
**Waiting list mortality**				
Programs, No.	30	56	28	
Marginal graft use rate^b^	13.9 (8.6)	15.9 (8.5)	17.7 (9.5)	.31
Waiting list mortality rate^c^	24.2 (6.7)	16.1 (2.0)	10.6 (1.7)	<.001
1-y graft and patient survival rate, %	90.5 (3.8)	90.0 (2.8)	90.6 (2.5)	.68
**Aggressiveness**				
Programs, No.	29	57	28	
Marginal graft use rate^b^	5.7 (2.6)	15.2 (2.8)	27.3 (7.5)	<.001
Waiting list mortality rate^c^	19.0 (8.0)	16.1 (5.0)	15.5 (5.5)	.05
1-y graft and patient survival rate, %	90.1 (3.4)	90.7 (2.4)	89.6 (3.5)	.27

^a^ *P* value generated with the Kruskal-Wallis test for nonparametric variables and the analysis of variance test for parametric variables.

^b^ Calculated as the rate of marginal grafts used per 100 transplants.

^c^ Calculated as the rate of death on the transplant waiting list per 100 person-years.

To investigate the correlation among each of these domains with the others, we generated scatterplots of the unadjusted rates for each of the 3 domains with each of the other domains (Figure). We found no significant correlation between a program’s 1-year graft and patient survival rate and its marginal graft use rate (β = −0.007; *P* = .83) or its waiting list mortality rate (β = −0.053; *P* = .19). Similarly, no correlation was found between each program’s waiting list mortality rate and its marginal graft use rate (β = −0.128; *P* = .06). We found a statistically significant unadjusted correlation between a program’s mean annual transplant volume and its marginal graft use rate (β = −0.011; *P* < .001).

**Figure.**
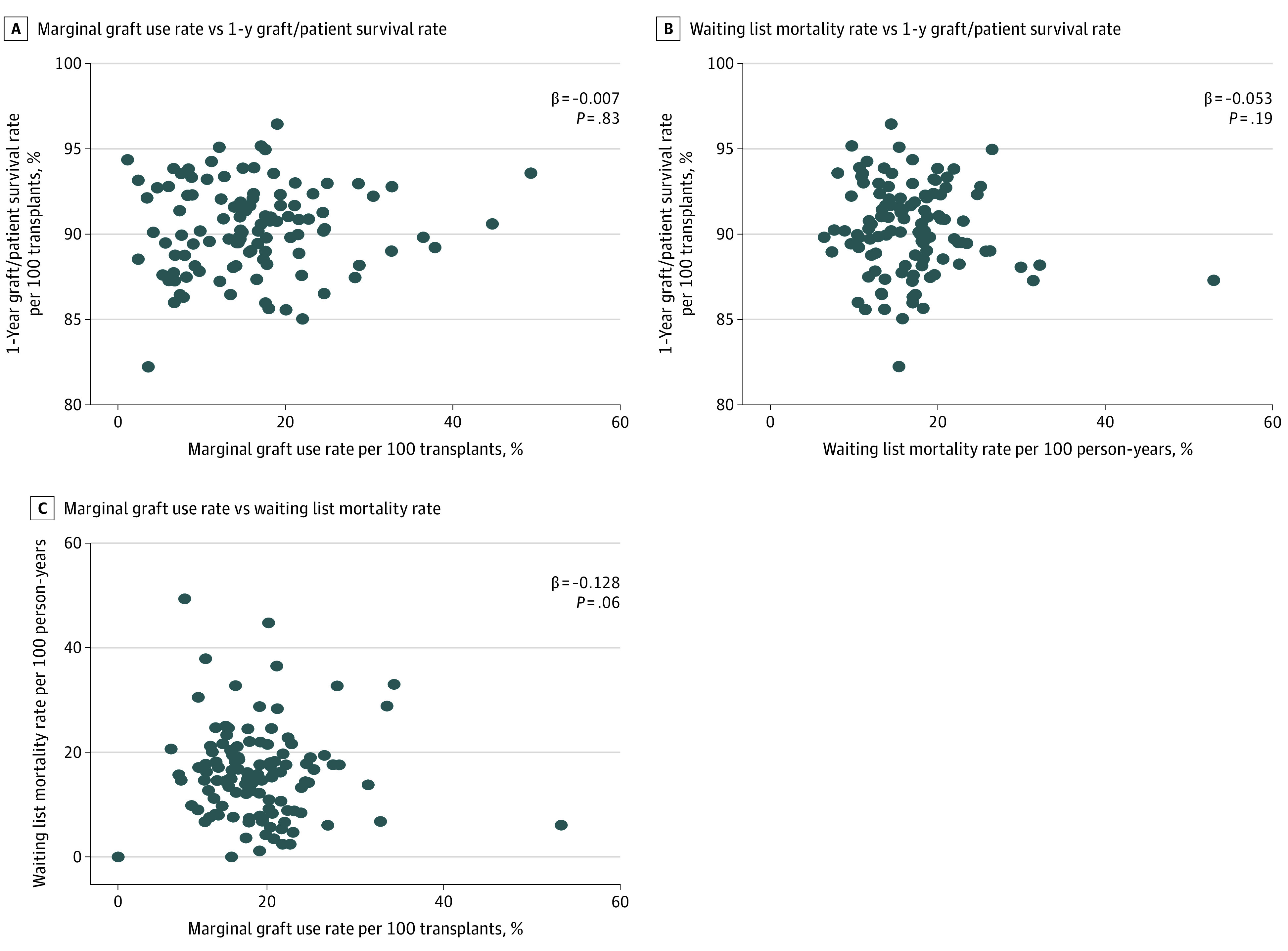
Scatterplots of Program-Level Measure Reported *P* values and correlation coefficients represents the results for the linear regression model between the 2 parameters controlling for mean MELD-Na at allocation.

The results of the additional analysis split by high- and low-competition DSA’s were similar to those found within the primary analysis. We found no significant correlations among a program’s 1-year graft and patient survival rate, marginal graft use rate, or its waiting list mortality rate, regardless of the transplant center being geographically located within a high- or low-competition DSA (eFigure 2 and 3 in the Supplement).

### Identification of High- and Low-Performing Programs

eFigure 4 in the Supplement shows the overlap among the 3 domains of quality investigated. We found that only 2 programs (1.8%) were included in the top quartile of all 3 domains. Six programs (5.2%) were in the top quartile for both marginal graft use and waiting list mortality but not 1-year graft and patient survival, whereas 5 programs (4.4%) were in the top quartile for both waiting list mortality and 1-year graft and patient survival but not marginal graft use, and another 4 programs (3.5%) were in the top quartile for both 1-year graft and patient survival and marginal graft use but not waiting list mortality. Finally, 15 programs (13.2%) were in only the top quartile for marginal graft use, 14 programs (12.3%) were only in the top quartile for waiting list mortality, and 15 programs (13.2%) were only in the top quartile for 1-year graft and patient survival. The remaining 50 programs (43.9%) were not in the top quartile for any of the 3 measures investigated. Similar proportions were found when we investigated the overlap among the bottom quartiles for the 3 domains of quality, with 4 programs (3.5%) representing the bottom quartile for all 3 domains (eFigure 5 in the Supplement).

## Discussion

In this cohort study of adult liver transplant programs in the US from 2014 through 2019, there was little variation in graft and patient survival rates compared with significantly more variation in waiting list mortality rates and use of marginal grafts. There was no correlation at the program level among any of the potential domains of quality. This remained true even within subgroups of DSA competition. There was a statistically significant but clinically insignificant association between transplant center volume and aggressiveness. Finally, we found that most programs achieved higher quality in 1 or even 2 domains but rarely in all 3 concurrently.

Current discussions of transplant program quality are often centered on recipient outcomes. The traditional measure within this domain, graft and patient survival, has several attributes limiting its utility as a quality measure. Specifically, 1-year graft and patient survival does not substantially vary across programs. This is exemplified in our analysis, in which survival rates for the programs investigated were highly clustered around the mean. In fact, median survival for programs in the top and bottom quartiles differed by only 5.8%. This results in a measure that is unable to meaningfully discriminate low-performing from high-performing programs. The measures investigated for the other 2 domains in this study, waiting list mortality rate and marginal graft use rate, were significantly more variable across programs and allowed for a greater ability to distinguish performance levels between institutions. Furthermore, measurement of the parameters required to generate data regarding these measures are already collected and would be easy to implement broadly, assuming the community agrees on their validity.

In a field where organs are a limited resource and competition is the norm, transplant programs and regulatory bodies have not traditionally emphasized the identification of programs that perform well across multiple domains of quality. Rather, the Final Rule’s establishment in 2007 of minimum standards for 1-year graft and patient survival^6^ made it clear that the emphasis had been placed on the use of recipient outcomes to find programs that perform poorly for review or sanctioning rather than to find programs that are excelling. We found that most programs in the bottom quartile for performance in recipient outcomes did not simultaneously perform poorly on measures of waiting list management and program aggressiveness. Particular emphasis on this single dimension of quality may unfairly penalize programs that perform better in other domains. It may also incentivize them to implement practices designed to improve recipient outcomes at the expense of other important domains of quality. The small number of centers identified in this study as high performers across all 3 domains represent an opportunity for the field of transplantation to learn from their outcomes, identify best practices, and eventually disseminate new strategies for quality improvement throughout the country and world.

Liver transplant programs may make changes to their practices under the assumption that a relationship exists among performance in the domains of waiting list management, aggressiveness, and recipient outcomes.^7,8^ For example, while promoting program aggressiveness may increase the number of transplants and therefore decrease waiting list mortality (assuming the waiting list does not change), using lower-quality grafts may result in a worsening of 1-year graft and patient survival.^9^ In an effort to improve survival rates, programs may elect not to place patients who are particularly ill on the waiting list in favor of recipients with increased likelihood of survival or may elect to instead pass on marginal grafts that they believe may have increased probability of failure compared with standard-risk organs.^10,11,12^ These strategies may worsen existing disparities in end-stage liver disease outcomes by limiting access to waiting list registration for patients receiving care in locations with poor performance on traditional survival measures. However, contrary to our hypotheses, we found that a program’s performance in any domain of quality was uncorrelated with its performance in the other domains. This has several important implications. First, the lack of correlation between program aggressiveness and 1-year graft and patient survival raises concerns about the principles guiding our decision-making around marginal grafts and the quality or usability of currently discarded grafts. Second, the lack of correlation between marginal graft use rate and waiting list mortality rate suggests that measurement of outcomes within the broad population of patients with liver disease is likely complex and at the very least our understanding of the factors affecting these measures are incomplete. For these reasons, performance in any 1 of or even several of these quality domains should be deemphasized in favor of a broader few of transplant center quality. Specifically, transplant centers should be incentivized to make certain their candidate pool reflects societal need, and, importantly, the demographic characteristics of the communities they serve. Transplant payer networks, including Centers for Medicare & Medicaid Services, should be asked to ensure that access to transplant is not overly cumbersome for marginalized populations, such as non–English speaking patients, members of racial/ethnic minority groups, and patients without financial resources. While payers have more influence over access to transplantation, center-level efforts to increase transplant access (eg, outreach and satellite clinics, increased social services, enhanced community engagement) should be incentivized and measured as factors associated with transplant center quality. Some measure of performance in the treatment of the broader population of liver disease seems particularly salient to our patients and the expansion of our measures of aggressiveness and waiting list outcomes to include components that more accurately reflect population-level quality may more closely approximate the ideal of true population-based quality measures.

### Limitations

The results of this study should be interpreted within the context of certain limitations. Our data represent program-level aggregated rates rather than rates calculated using individual patient data owing to restrictions from the SRTR on the use of center-identified patient-level data. While there was little variation in unadjusted rates of 1-year graft and patient survival, future studies using individual patient data allowing for adjustment for patient characteristics may reveal an association not seen with our analyses. Additionally, we used mean rates over a 5-year study period. It is possible that significant changes occurred over the 5 years of data included in this study such that a correlation may be present year-over-year that would be obscured by using longer time interval mean rates. That said, low annual volumes for many of the programs included could make shorter time scales less reliable as true measures of transplant program quality and more influenced by random variations in measure performance. This is further related to the cross-sectional nature of the study, limiting our ability to investigate how changes in performance in one domain of quality may be related to changes in another domain for a particular program. Additionally, our data do not contain information that may influence complexity of care (eg, patient demographics, comorbidities, or organ characteristics) and therefore limit our ability to account for the influence of these factors on program quality. Similarly, a variety of unmeasured center, region, organ-procurement organization, or donor service area factors may influence the association between any of the measured discussed herein. While it seems unlikely that these characteristics would differentially affect quality measure associations across centers, we cannot assess this directly in this study. We corrected for mean allocation MELD-Na in our models to adjust for known program-level differences in patient risk as well as a crude measure of donor availability, which may impact the quality measures assessed here, but further adjustment for clinical characteristics may reveal an association not elucidated in this study.

## Conclusions

This cohort study found that there was no correlation among adult liver transplant programs’ performance with respect to unadjusted 1-year graft and patient survival rates and performance on 2 alternative measures: waiting list mortality rates and marginal graft use rates. We also found that only 1.8% of programs were in the top quartile for all 3 measures, with the plurality of programs not represented in the top quartile for any of the 3 measures. These findings suggest that institutional-, regional-, or policy-level interventions to improve program performance on one measure may not have deleterious effects on performance on the other measures. However, further studies using individual patient-level data allowing for robust adjustment of clinical characteristics are needed.

## Notes

Supplement. **eFigure 1.** Scatterplot of Center-Level Mean Annual Transplant Volume vs Marginal Graft Use Rate per 100 Transplant Episodes **eFigure 2.** Scatterplots of Program Level Measure for Centers Within Low-Competition DSAs **eFigure 3.** Scatterplots of Program Level Measure Within High-Competition DSAs **eFigure 4.** Venn Diagram for the Top Performers in Each of the 3 Domains of Quality **eFigure 5.** Venn Diagram for the Bottom Performers for Each of the 3 Domains of Quality

## References

[1] Edwards E. 1991 Center Specific Graft & Patient Survival Rates. Department of Health and Human Services; 1992.

[2] Scientific Registry of Transplant Recipients. Program-specific reports: program-specific statistics on organ transplants. Accessed August 21, 2020. https://www.srtr.org/reports/program-specific-reports/10.1097/MOT.0000000000000597 30575617

[3] Garonzik-Wang JM, James NT, Arendonk KJV, . The aggressive phenotype revisited: utilization of higher-risk liver allografts. Am J Transplant. 2013;13(4):936-942. doi:10.1111/ajt.12151 23414232

[4] Organ Procurement and Transplantation Network. Proposal to implement pre-transplant performance review by the Membership and Professional Standards Committee. Accessed August 21, 2020. https://optn.transplant.hrsa.gov/media/1124/11_mpsc_pre_transplant_performance.pdf

[5] Sapiano MRP, Jones JM, Bowman J, Levi ME, Basavaraju SV. Impact of US Public Health Service increased risk deceased donor designation on organ utilization. Am J Transplant. 2019;19(9):2560-2569. doi:10.1111/ajt.15388 30959569 PMC6864734

[6] Centers for Medicare & Medicaid Services (CMS), HHS. Medicare program; hospital conditions of participation: requirements for approval and re-approval of transplant centers to perform organ transplants: final rule. Fed Regist. 2007;72(61):15197-15280. 17450666

[7] Mikolajczyk AE, Rao VL, Diaz GC, Renz JF. Can reporting more lead to less: the role of metrics in assessing liver transplant program performance. Clin Transplant. 2019;33(1):e13385. doi:10.1111/ctr.13385 30666739

[8] Chandraker A, Andreoni KA, Gaston RS, ; AST/ASTS Transplant Metrics Taskforce. Time for reform in transplant program-specific reporting: AST/ASTS transplant metrics taskforce. Am J Transplant. 2019;19(7):1888-1895. doi:10.1111/ajt.15394 31012525

[9] Lai JC, Feng S. Too aggressive or not aggressive enough: should a metric change center practice? Am J Transplant. 2013;13(4):837-838. doi:10.1111/ajt.12152 23551630 PMC3676686

[10] OPTN/UNOS Ethics Committee. Public comment proposal: manipulation of the waitlist priority of the organ allocation system through the escalation of medical therapies. Accessed August 31, 2020. https://optn.transplant.hrsa.gov/media/2380/ethics_publiccomment_20180122.pdf

[11] Snyder J. Gaming the liver transplant market. J Law Econ Organ. 2010;26(3):546-568. doi:10.1093/jleo/ewq003

[12] Dolgin NH, Movahedi B, Martins PNA, . Decade-long trends in liver transplant waitlist removal due to illness severity: the impact of centers for Medicare and Medicaid services policy. J Am Coll Surg. 2016;222(6):1054-1065. doi:10.1016/j.jamcollsurg.2016.03.021 27178368

