# Supplementary material for: Associations Among Different Domains of Quality Among US Liver Transplant Programs
Source: JAMA Netw Open. 2021 Aug 9;4(8):e2118502. doi: 10.1001/jamanetworkopen.2021.18502 (PMC8353538; doi:10.1001/jamanetworkopen.2021.18502)
Supplement: Supplement. — eFigure 1. Scatterplot of Center-Level Mean Annual Transplant Volume vs Marginal Graft Use Rate per 100 Transplant Episodes eFigure 2. Scatterplots of Program Level Measure for Centers Within Low-Competition DSAs eFigure 3. Scatterplots of Program Level Measure Within High-Competition DSAs eFigure 4. Venn Diagram for the Top Performers in Each of the 3 Domains of Quality eFigure 5. Venn Diagram for the Bottom Performers for Each of the 3 Domains of Quality [file jamanetwopen-e2118502-s001.pdf]

Supplemental Online Content

Brown CS, Waits SA, Englesbe MJ, Sonnenday CJ, Sheetz KH. Associations among different domains of quality among US liver transplant programs. *JAMA Netw Open*. 2021;4(8):e2118502. doi:10.1001/jamanetworkopen.2021.18502

eFigure 1. Scatterplot of Center-Level Mean Annual Transplant Volume vs Marginal Graft Use Rate per 100 Transplant Episodes

eFigure 2. Scatterplots of Program Level Measure for Centers Within Low-Competition DSAs

eFigure 3. Scatterplots of Program Level Measure Within High-Competition DSAs

eFigure 4. Venn Diagram for the Top Performers in Each of the 3 Domains of Quality

eFigure 5. Venn Diagram for the Bottom Performers for Each of the 3 Domains of Quality

This supplemental material has been provided by the authors to give readers additional information about their work.

eFigure 1. Scatterplot of Center-Level Mean Annual Transplant Volume vs Marginal Graft Use Rate per 100 Transplant Episodes

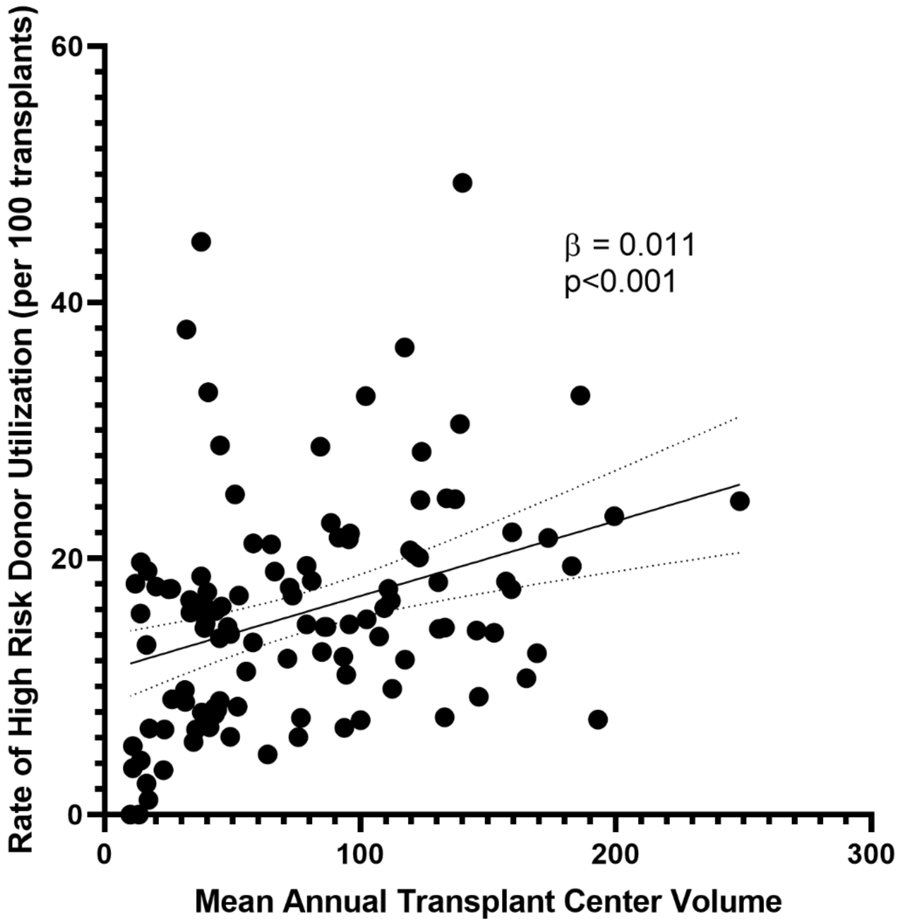

© 2021 Brown CS et al. *JAMA Network Open.*

eFigure 2. Scatterplots of Program Level Measure for Centers Within Low-Competition DSAs

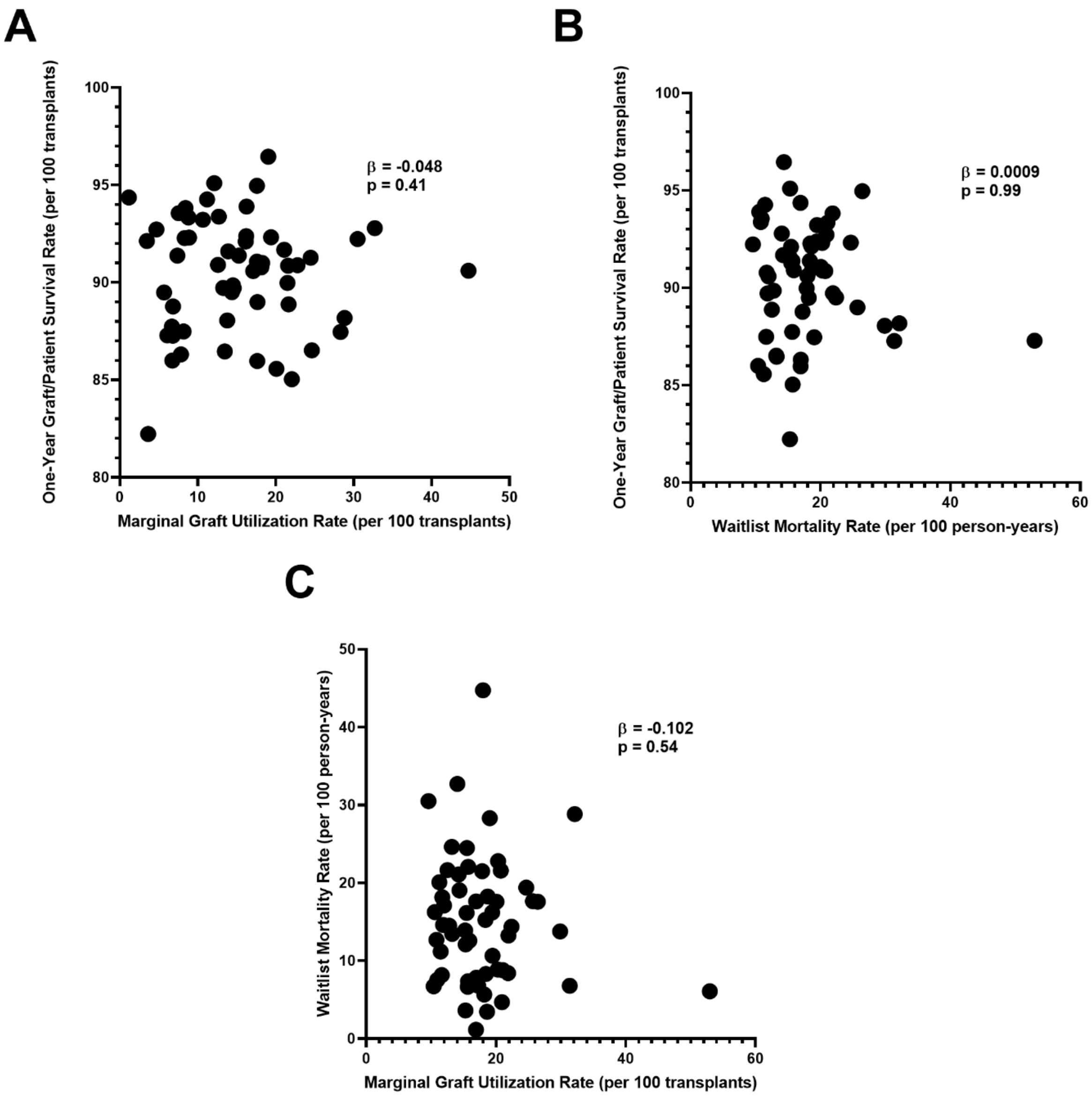

(A) marginal graft utilization rate vs one year graft/patient survival rate (B) waitlist mortality rate vs one year graft/patient survival rate (C) marginal graft utilization rate vs waitlist mortality rate. Reported p-values and correlation coefficients represents the results for the linear regression model between the two parameters controlling for mean MELD-Na at allocation.

© 2021 Brown CS et al. *JAMA Network Open.*

eFigure 3. Scatterplots of Program Level Measure Within High-Competition DSAs

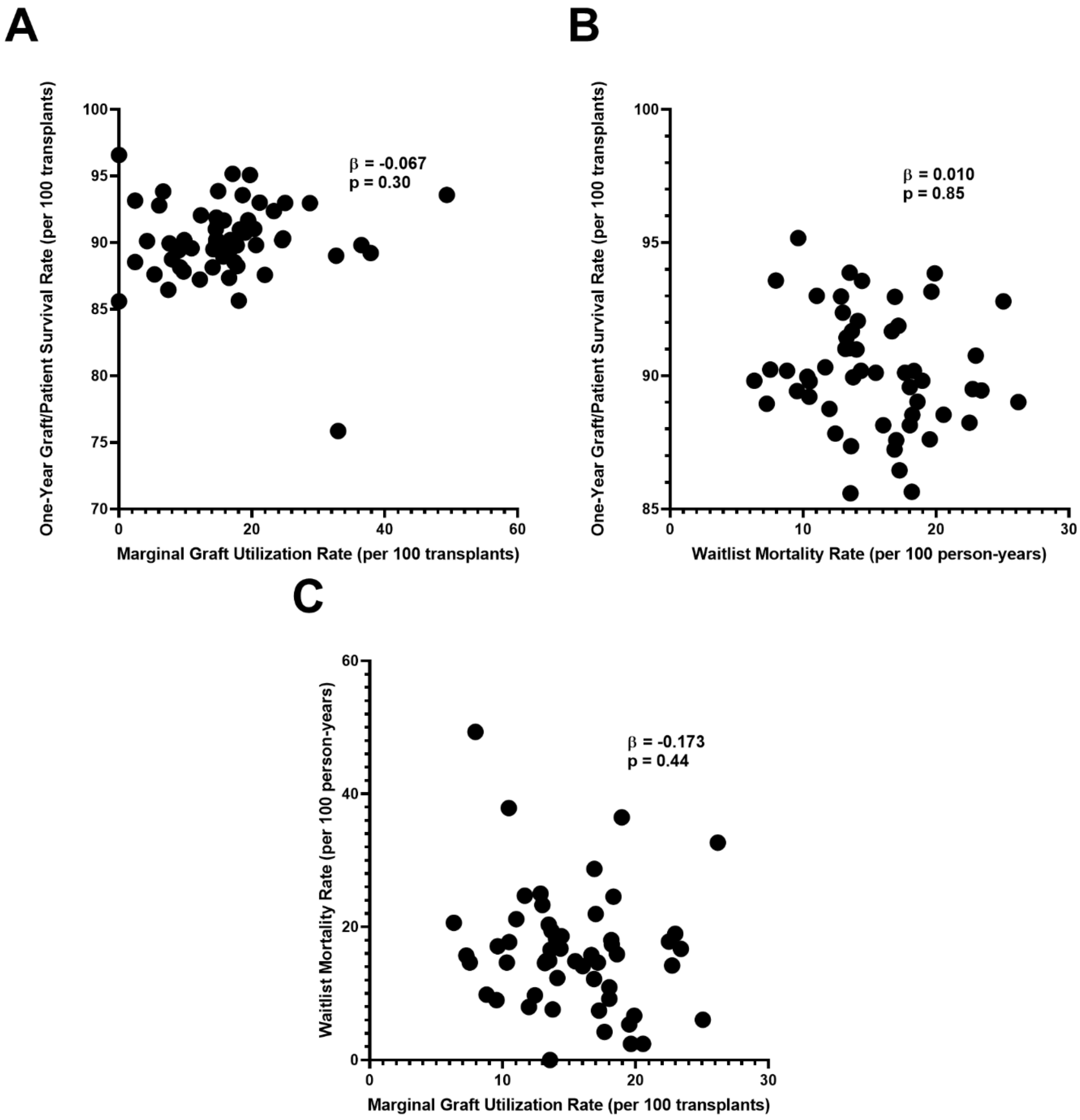

(A) marginal graft utilization rate vs one year graft/patient survival rate (B) waitlist mortality rate vs one year graft/patient survival rate (C) marginal graft utilization rate vs waitlist mortality rate. Reported p-values and correlation coefficients represents the results for the linear regression model between the two parameters controlling for mean MELD-Na at allocation.

© 2021 Brown CS et al. *JAMA Network Open.*

eFigure 4. Venn Diagram for the Top Performers in Each of the 3 Domains of Quality

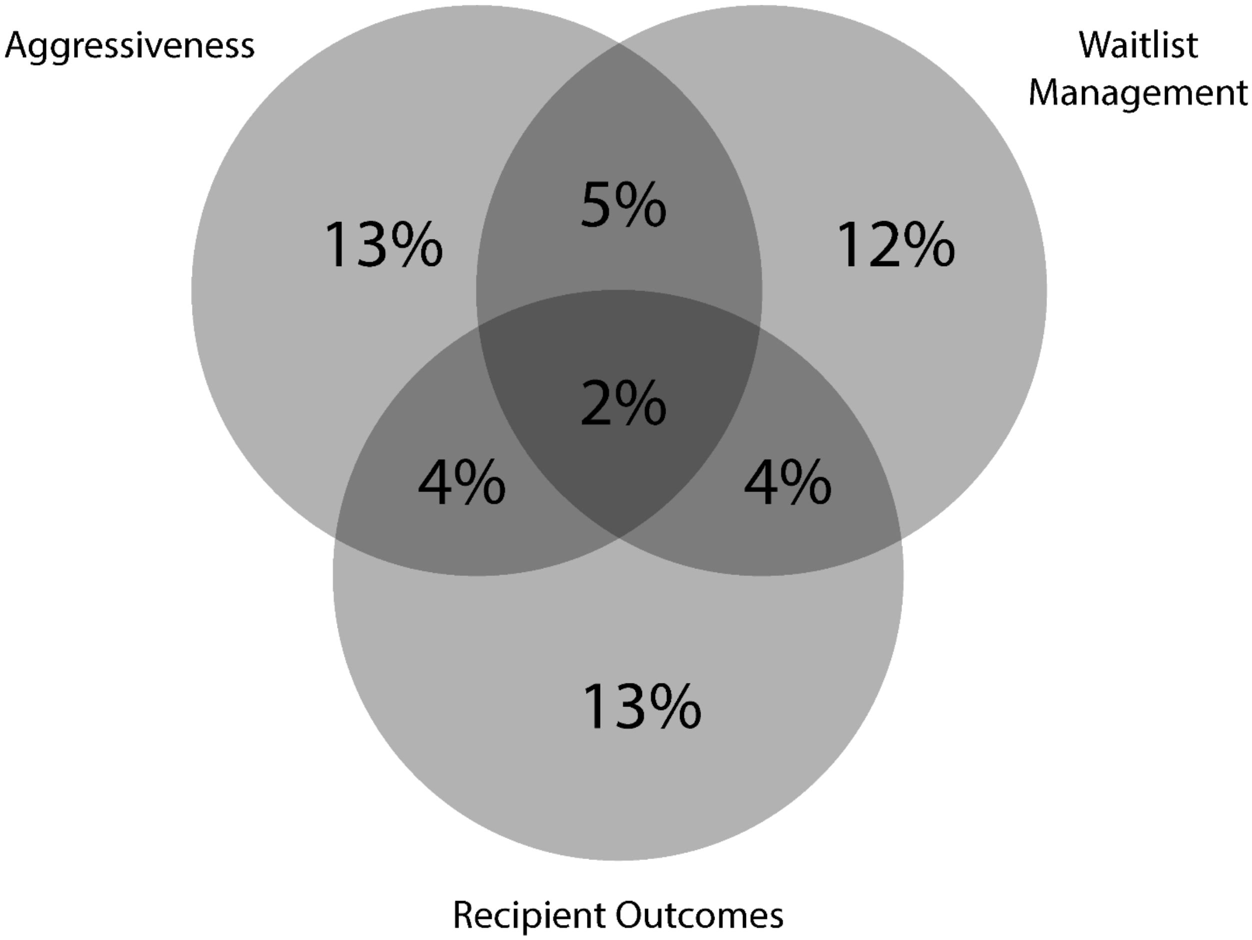

The area inside each circle represents the top quartile of programs for that domain of quality. Percentage values within each section of the diagram represent proportions of the total population of programs. Values are rounded to whole percentages.

© 2021 Brown CS et al. *JAMA Network Open.*

eFigure 5. Venn Diagram for the Bottom Performers for Each of the 3 Domains of Quality

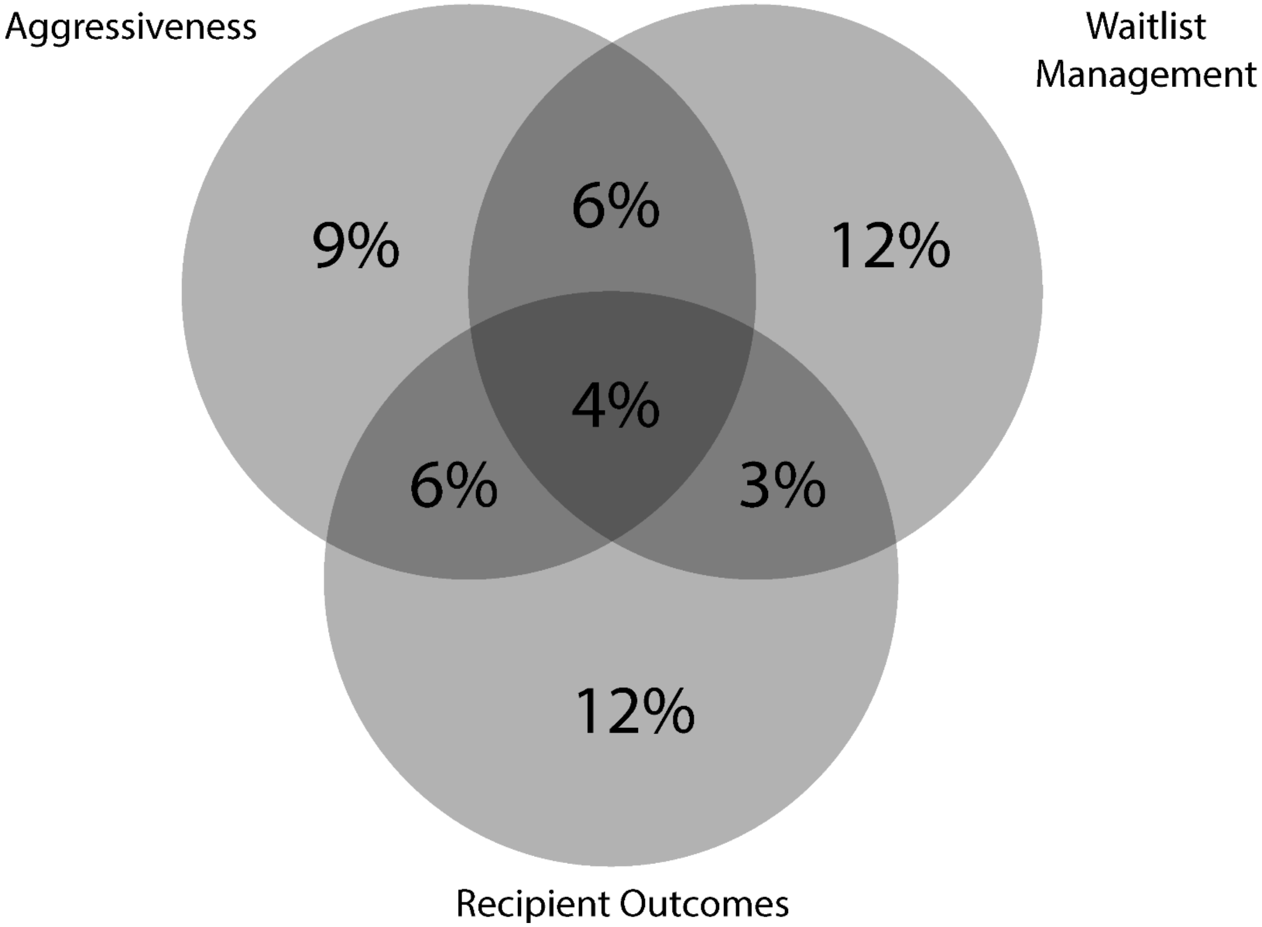

The area inside each circle represents the bottom quartile of programs for that domain of quality. Percentage values within each section of the diagram represent proportions of the total population of programs. Values are rounded to whole percentages.

© 2021 Brown CS et al. *JAMA Network Open.*
